# Racial/Ethnic Disparities in Antimicrobial Drug Use, United States, 2014–2015

**DOI:** 10.3201/eid2507.181775

**Published:** 2019-07

**Authors:** Mark Thomas, Naomi Whyler, Andrew Tomlin, Murray Tilyard

**Affiliations:** University of Auckland, Auckland, New Zealand (M. Thomas);; Auckland City Hospital, Auckland (M. Thomas, N. Whyler);; Best Practice Advocacy Centre, Dunedin, New Zealand (A. Tomlin, M. Tilyard)

**Keywords:** Antimicrobial drug use, race/ethnicity, antimicrobial resistance, New Zealand, United States, bacteria

**To the Editor:** We read with interest the article by Olesen and Grad (*1*), which reported that, in the United States during 2014–2015, the rate of antimicrobial drug use by white persons was twice that of persons of other races. The authors did not relate this finding to previous reports of ≈2 times lower incidence of sepsis (*2*) and ≈1.5 times lower incidence of death from infectious diseases (*3*) in white persons in the United States.

A national study of community antibacterial dispensing in relation to ethnicity in New Zealand (*4*) found that the dispensing rates were highest in Pacific people and Maori, consistent with their higher incidence of infectious diseases. However, the ethnic disparities in dispensing rates were substantially less than the ethnic disparities in the incidence of some infections. For example, even though the incidence of hospitalization for rheumatic fever was 63 times higher for Pacific people and 27 times higher for Maori than for persons of all other ethnicities combined, the annual dispensing rates of penicillins for Pacific people and Maori were <1.5 times higher than in other ethnicities.

Olesen and Grad suggest that ethnic disparities in antimicrobial drug use will lead to disparities in the prevalence of colonization (and disease) by antimicrobial-resistant bacteria. The New Zealand study found that dispensing rates of topical antimicrobial agents (predominantly fusidic acid) for Pacific and Maori children were approximately twice those for children of other ethnicities and suggested that these high dispensing rates might have contributed to the higher proportion of staphylococcal infections caused by methicillin-resistant (and fusidic acid–resistant) *Staphylococcus aureus* in Pacific people and Maori (*5*). We suggest that improved understanding of ethnic disparities in the incidence of infectious diseases and in the level of consumption of antimicrobial agents might usefully inform antimicrobial stewardship targets and strategies.
